# The Education in Aging and Geroscience Research (EAGR) Program for Undergraduate Students

**DOI:** 10.1093/geroni/igaf122.3856

**Published:** 2025-12-31

**Authors:** Kenneth Campellone, Rosie Mirabella, Iman Al-Naggar, Lisa Nigro

**Affiliations:** University of Connecticut, Storrs, Connecticut, United States; University of Connecticut, Storrs, Connecticut, United States; UConn Health, Farmington, Connecticut, United States; Central Connecticut State University, New Britain, Connecticut, United States

## Abstract

Aging is the greatest risk factor for most chronic illnesses including cardiovascular diseases, cancer, diabetes, and neurodegenerative disorders. As our population ages, the personal, professional, and economic burdens that accompany age-associated conditions continue to grow. The interdisciplinary field of geroscience aims to understand and manipulate the mechanisms of biological aging that impact the development of chronic diseases with the goal of improving human healthspan. While this concept is gaining traction in some graduate programs and medical schools, geroscience is not well recognized at the undergraduate level. To address the need for growing the geroscience workforce, the UConn Education in Aging and Geroscience Research (EAGR) Program provides teaching and training to the most foundational population for strengthening the geroscientist pipeline – undergraduate students. Our EAGR Program (R25 AG083251) provides a holistic multi-campus experience in which students are: (1) instructed in the breadth and depth of geroscience concepts through a customizable literature-based coursework track consisting of content, writing, and seminar classes; (2) trained in modern experimental approaches from basic to translational geroscience during a 2-week methods workshop with the themes of ‘Molecules-to-Cells’ and ‘Flies-to-Humans’; (3) immersed in geroscience research through individualized laboratory experiences in the summer and academic year; and (4) enabled to hone their communication skills and preparedness for advanced graduate, medical, or professional training. The structure, content, and outcomes from the first two years of the UConn EAGR program can help guide the development of similar geroscience training programs across other universities and medical school campuses.

